# Supplementary material for: Dynamics of local productive arrangements in the municipalities of Mato Grosso do Sul considering the transformations of the Bioceanic Corridor
Source: PLoS One. 2023 Apr 7;18(4):e0284023. doi: 10.1371/journal.pone.0284023 (PMC10081740; doi:10.1371/journal.pone.0284023)
Supplement: Attachment — Submitted filename: Response to Reviewers.docx [file pone.0284023.s001.docx]

**Response to Reviewers**

Thank you for appreciating our paper entitled “Dynamics of local productive arrangements in the municipalities of Mato Grosso do Sul considering the transformations of the Bioceanic Corridor”. Please, see below our responses to the reviewer’s comments. The changes made in the manuscript are highlighted in blue color. We emphasize that all suggestions were accepted.

**Database and free copyright images**

The data used in the research were obtained from public primary sources of the Brazilian federal government, publicly available online. These refer to information from the Annual Social Information Report (RAIS), a self-declaration that all companies (legal entities) must submit to the federal government (Ministry of Labor) at the end of each year.

Information on the primary database can be found at the link: <http://pdet.mte.gov.br/rais>. Specifically, the microdata are also available via an FTP server, via the link: <ftp://ftp.mtps.gov.br/pdet/microdados/>.

Another possibility of consultation would be through the BI provided by the Ministry of Labor, available at: <https://bi.mte.gov.br/bgcaged/login.php>. In this space, any user can use the login, “basico”, and password, “12345678”, to access (such login and password information are public available by the Ministry of Labor of Brazil).

With the primary public information data, we filtered the information for the spatial and temporal subset that we used in the research and performed the calculation of the spatial indices. Such a database elaborated in our research is available in the Mendeley database repository, available for public access at the link: <https://data.mendeley.com/datasets/bjf5f5zkwh>, DOI:10.17632/bjf5f5zkwh.2.

We drew up Figures 6, 7, 8, 9, and 10 combining the indicators calculated and presented in the methodology with a territorial mesh of the municipalities made available by IBGE - Brazilian Institute of Geography and Statistics through the website (<https://www.ibge.gov.br/geociencias/organizacao-do-territorio/malhas-territoriais/15774-malhas.html>). This website is publicly available as well as the data used in the territorial meshes. The software used to create the figures is QGIS - Geographic Information Systems based, open-source, and free to be downloaded and used by any researcher or student (<https://www.qgis.org/en/site/>). The software version used was 3.26.3 (Bucharest). Regarding all the figures in the paper, they are licensed by the authors under CC BY 4.0 License.

In addition, the data used in the preparation of the maps were obtained from public sources of the Brazilian federal government, available online and free access. These refer to the continuous cartographic base 1: 250000, version 2021, provided by (Brazilian Institute of Geography and Statistics) and the image of the CBERS-4A satellite of month 06 of 2022, made available by (National Institute for Space Research). Information about the Cartographic Base can be found at the link: <https://www.ibge.gov.br/geociencias/cartas-e-mapas/bases-cartograficas-continuas/15759-brasil.html?=&t=acesso-ao-produto> . The products of the Brazilian Institute of Geography and Statistics can be downloaded through the link: <https://www.ibge.gov.br/geociencias/downloads-geociencias.html> .

Information on the policies for the use of CBERS Satellite Images can be accessed at the link: <https://www.gov.br/inpe/pt-br/acesso-a-informacao/dados-abertos/> . The CBERS-4A satellite products made available by INPE can be accessed through the link: <http://www2.dgi.inpe.br/catalogo/explore> . All products used for the preparation of maps are available by the Brazilian government freely for research use, observed the citation of the source.

Reviewer #1: Abstract - Result, research outcome and contribution should be addressed. Abstract should be revised.

Introduction - Suggest to rewrite as it is identical to abstract.

Line 66-67 - Repetition, with intro and abstract.

Content, methodology, data analysis and final results are well presented with very minor flaw. Suggest to make a final proofread as typo found.

The abstract was elaborated again taking into account the suggestions. The introduction and repetition of line 66-67 has been rewritten. A typing review was performed.

Reviewer #2: The paper discussed the dynamics of local productive arrangements in the municipalities of Mato Grosso do Sul.The topic of this study is interesting.

(1)The abstract can be further refined. For example, the research results in the abstract can be summarized into three points.

(2)It is suggested to further improve the innovation points.

（3）The overall quality of English is good, but need to be checked carefully again. I suggest the authors should look for an English native speaker to further check the language of the paper.

（4）Some fresh paper should be added, like:

Sun, H., Samuel, C.A, Amissah, JCK, Taghizadeh-Hesary, F., Mensah, IA., 2020. Non-linear nexus between CO2 emissions and economic growth: A comparison of OECD and B&R countries, Energy, 212, 118637. <https://doi.org/10.1016/j.energy.2020.118637>.

The abstract was redone and highlighted the innovations and results. In the introduction, emphasis was placed on research innovations. The research was revised by a specialized company with native speakers, as can be seen in the certificate included to the resubmission. The research suggestion was included.
